# Urban and Rural Mpox Incidence Among Persons Aged 15–64 Years — United States, May 10–December 31, 2022

**DOI:** 10.15585/mmwr.mm7221a2

**Published:** 2023-05-26

**Authors:** Carla E. Zelaya, Brandi P. Smith, Aspen P. Riser, Jaeyoung Hong, Samantha Distler, Siobhán O’Connor, Ermias Belay, Mohammad Shoeb, Michelle A. Waltenburg, Maria E. Negron, Sascha Ellington

**Affiliations:** 1 CDC Mpox Emergency Response Team.

During May 10–December 31, 2022, a total of 29,980 confirmed and probable^†^ U.S. monkeypox (mpox) cases were reported to CDC, predominantly in cisgender adult men reporting recent same-gender sexual partners (*1*). Urban-rural differences in health (*2*) and diagnosis of HIV (*3**,**4*) and other sexually transmitted infections (*5*) are well documented nationally. This report describes urban-rural differences in mpox incidence (cases per 100,000 population) among persons aged 15–64 years, by gender and race and ethnicity. Urbanicity was assessed using the 2013 National Center for Health Statistics (NCHS) Urban-Rural Classification Scheme for Counties (*2*). Substantial differences in incidence by urbanicity, gender, and race and ethnicity were observed; most (71.0%) cases occurred in persons residing in large central urban areas. Among the cases in large central urban areas, most (95.7%) were in cisgender men. The overall incidence of mpox in the United States was 13.5 per 100,000 persons aged 15–64 years and peaked in August in both urban and rural areas. Among cisgender men, incidence in rural areas was approximately 4% that in large central urban areas (risk ratio [RR] = 0.04). Among cisgender women, incidence in rural areas was approximately 11% that in large central urban areas (RR = 0.11). In both urban and rural areas, incidence among non-Hispanic Black or African American (Black) and Hispanic or Latino (Hispanic) persons was consistently higher than that among non-Hispanic White (White) persons; RRs between Black and White persons were highest in rural areas. Support and maintenance of mpox surveillance and prevention efforts including vaccinations should focus on urban areas with the highest incidence of mpox during the 2022 outbreak; however, surveillance and prevention efforts should include all genders, persons of color, and persons residing in both urban and rural areas who are at increased risk for mpox.

Jurisdictional health departments electronically reported data on confirmed and probable mpox cases as part of the national case surveillance through a standardized case report form or via the National Notifiable Diseases Surveillance System.^§^ Urbanicity of county of residence was defined using the NCHS six-level classification scheme: four levels for metropolitan (urban) counties (large central, large fringe, medium, and small) and two levels for nonmetropolitan (rural) counties (micropolitan and noncore)^¶^ (*2*). Two U.S. counties were not represented in the analyses because they were not assigned an NCHS urban-rural classification; however, neither of these counties reported cases in 2022. Because of small case numbers, micropolitan and noncore groups were combined into one rural group. Incidence by each of the five levels of urbanicity was calculated by 1) summing cases from counties of the same level of urbanicity (numerator) and 2) summing county-level 2021 CDC WONDER population for persons aged 15–64 years** from counties with the same level of urbanicity (denominator). This analysis was limited to incidence estimates among persons aged 15–64 years because the majority of mpox cases were in this age range and to reduce bias that might be introduced by differential age distribution by urbanicity. Incidence estimates were stratified by month, gender, or race and ethnicity. RRs with 95% CIs were calculated between groups (e.g., urban compared with rural areas). This activity was reviewed by CDC and was conducted consistent with applicable federal law and CDC policy.^††^

The 29,980 confirmed and probable mpox cases reported to CDC in 2022 included 29,311 (97.8%) cases among persons aged 15–64 years in the 50 states and the District of Columbia. Most cases among persons aged 15–64 years occurred in large central urban areas (71.0%); however, cases were reported at each level of urbanicity, including 440 (1.5%) in rural areas (Table 1). The median age (34 years) and distribution of mpox patients by age group were similar for all urban-rural categories. In large central urban areas, 95.7% and 2.3% of cases were in cisgender men and cisgender women, respectively, and in rural areas, 94.7% and 4.6% of cases were in these groups, respectively.

**TABLE 1:** Demographic characteristics of persons aged 15–64 years with mpox,* by urban-rural classification^†^ of county of residence — United States,^§^ May 10–December 31, 2022

Characteristic (no. with available information)	Urban-rural classification, no. (%)						
	Overall^¶^ (N = 29,176)	Large central (n = 20,710)	Large fringe (n = 5,321)	Medium (n = 2,177)	Small (n = 528)	Nonmetro or rural (n = 440)	Missing county of residence (n = 135)
Age, median, yrs (IQR)	**34 (29–42)**	35 (29–42)	34 (28–41)	33 (27–40)	32 (26–40)	34 (27–41)	33 (28–40)
**Age group, yrs (29,176)**							
15–29	**7,939 (27.2)**	5,276 (25.5)	1,599 (30.1)	729 (33.5)	190 (36.0)	145 (33.0)	43 (31.9)
30–39	**12,156 (41.7)**	8,860 (42.8)	2,107 (39.6)	841 (38.6)	193 (36.6)	155 (35.2)	58 (43.0)
40–49	**6,010 (20.6)**	4,385 (21.2)	1,041 (19.6)	396 (18.2)	92 (17.4)	96 (21.8)	28 (20.7)
50–64	**3,071 (10.5)**	2,189 (10.6)	574 (10.8)	211 (9.7)	53 (10.0)	44 (10.0)	6 (4.4)
Gender** (28,941)							
Cisgender men	**27,597 (95.4)**	19,725 (95.7)	4,943 (94.9)	2,036 (93.7)	484 (92.9)	409 (94.7)	129 (97.7)
Cisgender women	**820 (2.8)**	475 (2.3)	195 (3.7)	101 (4.7)	29 (5.6)	20 (4.6)	2 (1.5)
Transgender men	**70 (0.2)**	42 (0.2)	14 (0.3)	9 (0.4)	4 (0.8)	1 (0.2)	1 (0.8)
Transgender women	**249 (0.9)**	191 (0.9)	38 (0.7)	16 (0.7)	2 (0.4)	2 (0.5)	0 (—)
Another sex or gender	**205 (0.7)**	173 (0.8)	20 (0.4)	10 (0.5)	2 (0.4)	0 (—)	0 (—)
**Race and ethnicity^††^ (27,503)**							
AI/AN	**112 (0.4)**	79 (0.4)	6 (0.1)	15 (0.7)	4 (0.8)	8 (1.9)	0 (—)
Asian	**779 (2.8)**	623 (3.2)	112 (2.2)	33 (1.6)	2 (0.4)	9 (2.1)	5 (6.2)
Black or African American	**9,151 (33.3)**	6,000 (30.8)	2,019 (40.1)	784 (37.8)	197 (39.2)	151 (35.1)	20 (24.7)
NH/OPI	**70 (0.3)**	48 (0.2)	8 (0.2)	12 (0.6)	0 (—)	2 (0.5)	0 (—)
White	**8,050 (29.3)**	5,487 (28.2)	1,421 (28.2)	741 (35.8)	215 (42.8)	186 (43.3)	29 (35.8)
Hispanic or Latino	**8,500 (30.9)**	6,584 (33.8)	1,334 (26.5)	445 (21.5)	72 (14.3)	65 (15.1)	26 (32.1)
Multiple or other races	**841 (3.1)**	642 (3.3)	136 (2.7)	42 (2.0)	12 (2.4)	9 (2.1)	1 (1.2)

**Abbreviations:** AI/AN = American Indian or Alaska Native; NH/OPI = Native Hawaiian or other Pacific Islander.

* Includes confirmed and probable cases among persons aged 15–64 years; data were updated as of January 31, 2023. Cases missing information on age (207) were not included.

^†^ Urban-rural classification of county of residence is based on the 2013 National Center for Health Statistics Urban-Rural Classification Scheme for Counties. The two nonmetro categories were combined into one rural (i.e., nonmetropolitan) category.

^§^ Counties from the 50 states and the District of Columbia reporting at least one resident aged 15–64 years with mpox are included. A total of 983 of 3,141 (31%) counties are represented in this table, and the percentages of counties represented by urbanicity were as follows: 100% in large central urban areas, 70% in large fringe urban areas, 59% in medium urban areas, 47% in small urban areas, and 14% in nonmetro or rural areas.

^¶^ Cases with missing information on county of residence (135) are not included in the overall category.

** Methods for collecting gender information are not standardized across all jurisdictions. When self-reported gender was missing, current sex or sex assigned at birth was used, and gender identity was presumed to be cisgender. Among the 29,311 cases reported in 2022, a total of 238 cases were missing data on gender (i.e., gender, sex, and sex assigned at birth).

^††^ All persons who reported Hispanic or Latino (Hispanic) ethnicity, regardless of race, were categorized as Hispanic. Persons who did not report ethnicity as Hispanic (including missing ethnicity) were categorized as non-Hispanic and reported race in the following categories: AI/AN, Asian, Black or African American, NH/OPI, White, and multiple races (more than one race category selected) or other race. Persons with missing data on race and ethnicity were categorized as missing or unknown.

Racial and ethnic distributions of cases differed by urbanicity. Hispanic persons accounted for 33.8% and 26.5% of cases in large central urban and large fringe urban areas, respectively, but only 14.3% and 15.1% in small urban and rural areas, respectively. The proportion of cases in small urban and rural areas among White persons (42.8% and 43.3% respectively), was higher than that in other urban areas (28.2% in large central, 28.2% in large fringe, and 35.8% in medium). The proportion of cases among Black persons differed across each category of urbanicity, ranging from 30.8% in large central urban areas to 40.1% in large fringe urban areas. The number of mpox cases in other racial and ethnic groups was small, and the relationship with urbanicity could not be assessed.

Overall mpox incidence was 13.5 per 100,000 (Table 2) but varied by urbanicity. Rates in large fringe urban areas (9.7), medium urban areas (4.9), small urban areas (2.8), and rural areas (1.5) were 32%, 16%, 9%, and 5% the rate in large central urban areas (30.6), respectively (Table 2) (Supplementary Table 1, https://stacks.cdc.gov/view/cdc/128431). In all areas, incidence peaked in August and then declined during October–December.

**TABLE 2:** Mpox incidence* among persons aged 15–64 years, by month^†^ and urban-rural classification^§^ of county of residence — United States, May 10–December 31, 2022

Urban-rural classification	Incidence, by month						Overall risk ratio (95% CI)**
	May–Jun	Jul^¶^	Aug^¶^	Sep^¶^	Oct–Dec^¶^	Overall	
**All areas**	**0.5**	**4.2**	**5.3**	**2.3**	**1.3**	**13.5**	—
**Urban**							
Large central urban	1.3	10.0	11.7	4.9	2.6	**30.6**	Ref
Large fringe urban	0.3	2.9	3.9	1.6	1.0	**9.7**	0.32 (0.31–0.33)
Medium urban	0.1	1.0	1.9	1.1	0.7	**4.9**	0.16 (0.15–0.17)
Small urban	0.1	0.6	1.0	0.7	0.5	**2.8**	0.09 (0.08–0.10)
**Rural**							
Nonmetropolitan	0	0.3	0.6	0.4	0.2	**1.5**	0.05 (0.05–0.06)

**Abbreviation**: Ref = reference group.

* Cases per 100,000 population, calculated using summed case counts and population size (persons aged 15–64 years) for all areas and for each level of urbanicity, multiplied by 100,000.

**^†^** Incidence during May–June and during October–December was combined because case counts were low during those periods.

^§^ Urban-rural classification of county of residence is based on the 2013 National Center for Health Statistics Urban-Rural Classification Scheme for Counties. The two nonmetro categories were combined into one rural (i.e., nonmetropolitan) category. Among the 29,311 cases reported in 2022, 135 were missing data on county of residence; therefore, incidences were calculated based on a total of 29,176 mpox cases.

^¶^ The number of cases in each period (numerator) was subtracted from the denominator for the following period when calculating incidence.

** Risk ratio was calculated by comparing large fringe urban, medium urban, small urban, and rural (nonmetropolitan) areas with large central urban areas.

Overall, incidence was 27.2 in cisgender men and 0.7 in cisgender women (Supplementary Table 2, https://stacks.cdc.gov/view/cdc/128432). Among both groups, rates were highest in large central urban areas and were lower in small urban areas (Figure), (Supplementary Table 1, https://stacks.cdc.gov/view/cdc/128431) (Supplementary Table 2, https://stacks.cdc.gov/view/cdc/128432). Among cisgender men, incidence in rural areas (2.8 per 100,000) was approximately 4% that in large central urban areas (65.0; RR = 0.04). Among cisgender women, rates in rural areas (0.1) were approximately 11% that in large central urban areas (1.3; RR = 0.11).

**FIGURE:**
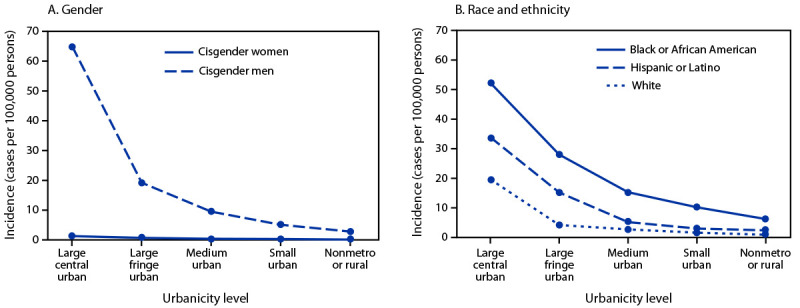
Mpox incidence* among persons aged 15–64 years, by gender^†^ (A), race and ethnicity^§^ (B), and urban-rural classification^¶^ of county of residence — United States, May 10–December 31, 2022 * Cases per 100,000 population. Risk was calculated by using summed case counts and population size (persons aged 15–64 years) by gender and race and ethnicity and each level of urban-rural classification, multiplied by 100,000. ^†^ Methods for collecting gender information are not standardized across all jurisdictions. When self-reported gender was missing, current sex or sex assigned at birth was used, and gender identity was presumed to be cisgender. Among the 29,311 cases reported in 2022, 238 cases were missing data on gender (i.e., gender, sex, and sex assigned at birth). Among cases reported in cisgender men (27,726), 129 were missing information on county of residence, and risk for cisgender men was calculated from a total of 27,597 mpox cases among cisgender men. Among cases reported in cisgender women (822), two were missing information on county of residence, and incidence among cisgender women was calculated from a total of 820 mpox cases. ^§^ All persons who reported Hispanic or Latino (Hispanic) ethnicity, regardless of race, were categorized as Hispanic. Persons who did not report ethnicity as Hispanic (including missing ethnicity) were categorized as non-Hispanic and reported race in the following categories: American Indian or Alaska Native, Asian, Black or African American (Black), Native Hawaiian or other Pacific Islander, White, and multiple races (more than one race category selected) or other race. This figure includes Black, Hispanic, and White racial and ethnic groups; incidence in all other groups was unreliable because of small sample sizes and were not included (https://stacks.cdc.gov/view/cdc/128433). Persons with missing data on ethnicity and race were categorized as missing or unknown. Among the 29,311 cases reported in 2022, 1,727 cases were missing data on race and ethnicity. Incidences among Black, Hispanic, and White persons were calculated using 9,151, 8,500, and 8,500 cases, respectively, because of the number of cases missing information on county of residence (20 of 9,171 [Black], 26 of 8,526 [Hispanic], and 29 of 8,079 [White]). ^¶^ Urban-rural classification of county of residence is based on the 2013 National Center for Health Statistics Urban-Rural Classification Scheme for Counties. The two nonmetro categories were combined into one rural (i.e., nonmetropolitan) category.

In urban and rural areas, incidence was higher among cisgender men than among cisgender women, with the highest RR (51.2) in large central urban areas where the incidence in cisgender men was highest (Supplementary Table 1, https://stacks.cdc.gov/view/cdc/128431). In rural areas, the relative difference in incidence between cisgender men and cisgender women was lower (RR = 19.8).

In both urban and rural areas, incidence was higher among Black persons compared with White persons (Figure) (Supplementary Table 3, https://stacks.cdc.gov/view/cdc/128433). The relative difference in incidence between Black and White persons was highest in rural areas (RR = 7.2) (Supplementary Table 1, https://stacks.cdc.gov/view/cdc/128431); however, the absolute difference was highest in large central urban areas where incidence per 100,000 persons aged 15–64 years in Black persons was 52.3 and in White persons was 19.7 (Supplementary Table 3, https://stacks.cdc.gov/view/cdc/128433). In addition, in urban and rural areas, rates were higher among Hispanic persons than among White persons (Figure). Lastly, in urban and rural areas, incidence was higher among Black persons than among Hispanic persons.

## Discussion

In the United States, 85% of the population lives in areas classified as urban (*2*), and the majority of mpox cases occurred in urban areas (*1*), with the highest incidence in large central urban areas. Across all urban-rural levels, incidence peaked in August 2022. Although the number of mpox cases decreased sharply after this date, the risk for outbreak and recurrence is dependent on the estimated immunity level, which is calculated by the number of cases during the 2022 outbreak and vaccination rates (*6*).

During the 2022 mpox outbreak, approximately 95% of mpox cases were among cisgender men. Among both cisgender men and cisgender women, incidence was lower with decreasing urbanicity. Incidence was consistently higher among cisgender men than among cisgender women in all urban-rural areas; however, the relative difference in incidence was less in rural areas.

In large central urban areas, proportions of cases among Black, Hispanic, and White persons were similar; however, the incidence was much higher among Black and Hispanic persons than among White persons. In rural areas, most cases were among both Black (35%) and White (43%) persons; however, rates among Black (6.2) and Hispanic (2.4) persons were approximately six and two times higher, respectively, than incidence among White persons (0.9).

During the multinational outbreak in 2022, 1- and 2- dose mpox vaccination coverage, among persons at risk in the United States, reached an estimated 37% and 23%, respectively, with wide variation across the country (*7*). Preventive efforts, including vaccination campaigns, were concentrated in urban areas because of the large number of cases in these areas during the mpox response (*7*). The estimated higher incidence in urban areas underscores the need for services, especially in large central urban areas; however, mpox prevention, recognition, diagnosis, and treatment services are still needed in less urban and rural areas. In addition, in light of the lower incidence observed in less urban areas and potentially lower levels of immunity acquired through natural infection, larger proportions of the population in smaller urban and rural areas might be susceptible to infection.

Previously published information indicated that barriers to accessing prevention services in rural areas might result from fewer service locations, shortages in providers, cultural barriers, stigma, and fewer provider referrals for testing and vaccination (*5*,*8*). Further, these barriers exist in the context of persistent racial and ethnic health disparities, particularly among men, in rural areas (*9*). In rural areas, the higher incidence observed among Black and Hispanic persons compared with White persons might be attributed to known barriers to accessing care combined with existing health inequalities. Further research into mpox risk factors, incidence, transmission potential, vaccination coverage, and access to prevention and care services by urbanicity, gender, and race and ethnicity is needed for public health decision-making and improving equitable prevention and care among those at risk. 

The findings in this report are subject to at least three limitations. First, sample sizes did not permit reliable estimation of incidence by urbanicity among transgender or gender diverse persons, or among some racial and ethnic groups. Second, methods for collecting gender information are not standardized across the United States. When self-reported gender data were missing, current sex or sex assigned at birth was used, and gender identity was presumed to be cisgender. This limitation could have resulted in undercounting transgender or gender-diverse persons, particularly in jurisdictions that do not routinely collect this information. Finally, persons with mpox might have been less likely to receive an mpox diagnosis in rural areas, potentially because of inadequate access or limited testing availability. If mpox was less likely to be detected and consequently diagnosed in less urban and rural areas, than in large central urban areas, incidence might have been underestimated in rural and less urban areas.

The 2022 U.S. mpox outbreak was largely driven by transmission among cisgender men reporting recent same-gender sexual partners in large central urban areas (*1*). However, although incidence was lower in less urban areas, this analysis found that cases also occurred among cisgender men in less urban areas, and among cisgender women in both urban and rural areas. Efforts to support and maintain mpox surveillance should be continued nationally in all areas of urbanicity to ensure all persons at risk for mpox get tested and treated. The current analysis demonstrated that racial and ethnic disparities in mpox incidence that were previously documented (*10*) were higher in absolute magnitude in urban areas and higher in relative magnitude in smaller urban and rural areas. This underscores the need for continued implementation of equity-based vaccination strategies focused on gay, bisexual, and other men who have sex with men (MSM) in urban areas where most mpox cases have been reported. In addition, comprehensive prevention strategies should include all persons at risk for mpox. CDC continues to recommend a full 2-dose course of the JYNNEOS vaccines for MSM and others at risk for *Monkeypox virus* exposure.

**Summary**

**What is already known about this topic?**

Monkeypox (mpox) has disproportionately affected gay, bisexual, and other men who have sex with men (MSM). Information on urbanicity of mpox cases during the 2022 outbreak is limited.

**What is added by this report?**

During May–December 2022, U.S. mpox incidence was 13.5 per 100,000 persons peaking in August. Among cisgender men and cisgender women, incidence in rural areas was 4% and 11% of incidence in large central urban areas, respectively. Incidence among Black or African American and Hispanic or Latino persons was higher than among White persons.

**What are the implications for public health practice?**

National mpox surveillance should be continued to ensure persons at risk for mpox get tested and treated. Prevention efforts should be focused on MSM in urban areas.

## Footnotes

* These authors contributed equally to this report.

† https://www.cdc.gov/poxvirus/mpox/health-departments/case-reporting.html (Accessed January 21, 2023).

§ https://www.cdc.gov/nndss/index.html (Accessed January 21, 2023).

¶
https://www.cdc.gov/nchs/data/data_acces_files/2014-rural-urban-chartbook-update.pdf

** Single-Race Population Estimates, United States, 2020–2021. July 1 resident population by state, county, age, sex, single-race, and Hispanic origin, on CDC WONDER. CDC county-level data are available in 5-year data increments. Vintage 2021 estimates were released by the U.S. Census Bureau on June 30, 2022. https://wonder.cdc.gov/single-race-v2021.html (Accessed December 27, 2022).

†† 45 C.F.R. part 46.102(l)(2), 21 C.F.R. part 56; 42 U.S.C. Sect. 241(d); 5 U.S.C. Sect. 552a; 44 U.S.C. Sect. 3501 et seq.
